# Functional Outcomes of Orthotopic Neobladder in Women

**DOI:** 10.1007/s11934-024-01223-7

**Published:** 2024-08-28

**Authors:** Unwanaobong Nseyo, David Ginsberg

**Affiliations:** 1 Department of Urology, Weill Cornell Medicine, New York, NY USA; 2 Department of Urology. Keck School of Medicine of USC, 1441 Eastlake Ave Suite 7416, Los Angeles, CA 90089 USA

**Keywords:** Orthotopic neobladder, Urinary incontinence, Urinary retention, Female, Radical cystectomy

## Abstract

**Purpose of Review:**

This review paper summarizes the available literature on the evolution of surgical approach to radical cystectomy in female bladder cancer patients and its impact on functional outcomes in orthotopic neobladder.

**Recent Findings:**

Traditionally, radical cystectomy in female bladder cancer patients has been maximally extirpative with pelvic exenteration. Recently, new techniques which include pelvic organ-sparing, nerve-sparing and vaginal-sparing have demonstrated improved rates of urinary incontinence and retention. Additional techniques include prophylactic apical suspension which reduces the likelihood of pelvic organ prolapse, a risk factor for voiding dysfunction in the setting of orthotopic neobladder.

**Summary:**

Surgical management of bladder cancer in female patients has evolved to include surgical approaches which center quality of life and functional outcomes that are unique to female patients who have undergone radical cystectomy with ileal neobladder and can be optimized based on considerations regarding an approach that limits pelvic floor and pelvic nerve disruption.

## Introduction

Bladder cancer is the tenth most common malignancy. It has a strong male predominance as men are three to four times more likely to be diagnosed with bladder cancer, with the incidence among females 75,000 cases worldwide per year [1]. Yet, despite a higher prevalence among males, female patients are more likely to have locally advanced disease at diagnosis [2, 3]. Additionally, women with bladder cancer have higher mortality at all stages which is thought to be due to delays in diagnosis [3–6].

With radical cystectomy the standard of care for muscle-invasive and high-risk non-muscle invasive disease, the higher stage at diagnosis for female patients means that they are more likely to require radical cystectomy for oncologic control [7, 8]. Concerns about the oncologic outcomes due to gender-based anatomical differences initially drove decisions about the surgical technique for radical cystectomy in women [9–11]. Classically, radical cystectomy in the female patient was maximally extirpative and included anterior exenteration with the removal of the anterior vagina, the uterus, and ovaries as well as pelvic lymph node dissection [12, 13]. The wide degree of resection was informed by concerns regarding micrometastatic disease and the risk for positive margins, as the rate of urethral recurrence was previously quoted to be as high as 36% [14]. However, recent pathological studies have confirmed the oncological safety of maintaining the female urethra and the decision to spare female pelvic organs and maximize urethral length at the time of radical cystectomy has followed [15–25]. Stein et al. performed a prospective analysis of female cystectomy patients and identified that 70% would be candidates for neobladder construction based on lower rates of local recurrence than previously thought and surgical options for managing muscle-invasive bladder cancer in the female patient have expanded as a result [16]. Anterior pelvic exenteration has been replaced in the appropriate patient with less extensive approaches, such as pelvic organ-sparing techniques, nerve-sparing, and urethral-sparing approaches [8, 17, 26, 27]. In a contemporary setting, additional techniques have been designed with the goal of balancing the maximization of oncologic outcomes with optimizing the quality of functional outcomes.

## Considerations for Use of Orthotopic Neobladder in Female Patients

While oncologic outcomes dictated the degree of resection, functional outcomes in the post-cystectomy female patient, such as urinary incontinence and voiding dysfunction, contributed to decision-making regarding diversion type at the time of radical cystectomy, which includes orthotopic or heterotopic and continent versus incontinent diversions. Historically, orthotopic neobladder (ONB) had been underutilized following radical cystectomy in female patients as post-cystectomy female urethral length was thought to be too short for functional continence [11, 28]. To compensate for the shortened urethra, complex lower urinary tract reconstruction was performed at the time of radical cystectomy which included placement of an omental flap between the neobladder and the vagina for support, utilization of a sigmoid neobladder, suturing of the neobladder to the pubic symphysis and augmenting the pelvic floor with mesh [22, 29–32].

An improved understanding of the female pelvic anatomy, specifically the rhabdosphincter, has facilitated the use of ONB in female bladder cancer patients. Anatomic studies have characterized the structure and innervation of the urethral rhabdosphincter as well as the musculature of the pelvic floor, further emphasizing the role of the female pelvic anatomy in determining functional outcomes [21, 33–37]. Advanced knowledge about the female urethra and pelvic anatomy has refuted the concern about the inadequacy of the post-radical cystectomy female urethra.

With oncologic safety validated and concerns regarding functional outcomes allayed, a frame shift occurred in which the focus turned towards survivorship concerns for women with bladder cancer who had undergone a radical cystectomy, which centered on issues of continence and sexual function. Early data on continence and sexual function after pelvic exenteration indicated that the extent of the surgical resection had a significant impact on the quality of life for women post-cystectomy [38–41]. Women who had undergone pelvic exenteration were less likely to be sexually active and more likely to report dyspareunia [12, 42, 43]. Additional quality of life concerns for female cystectomy patients were found to be closely related to voiding function and urinary symptoms, both of which were related to surgical approach [44–46]. Consideration of quality of life with respect to functional outcomes allowed for the development of surgical approaches that prioritized elements of these functional outcomes.

## Surgical Technique and Functional Outcomes

**Fig. 1:**
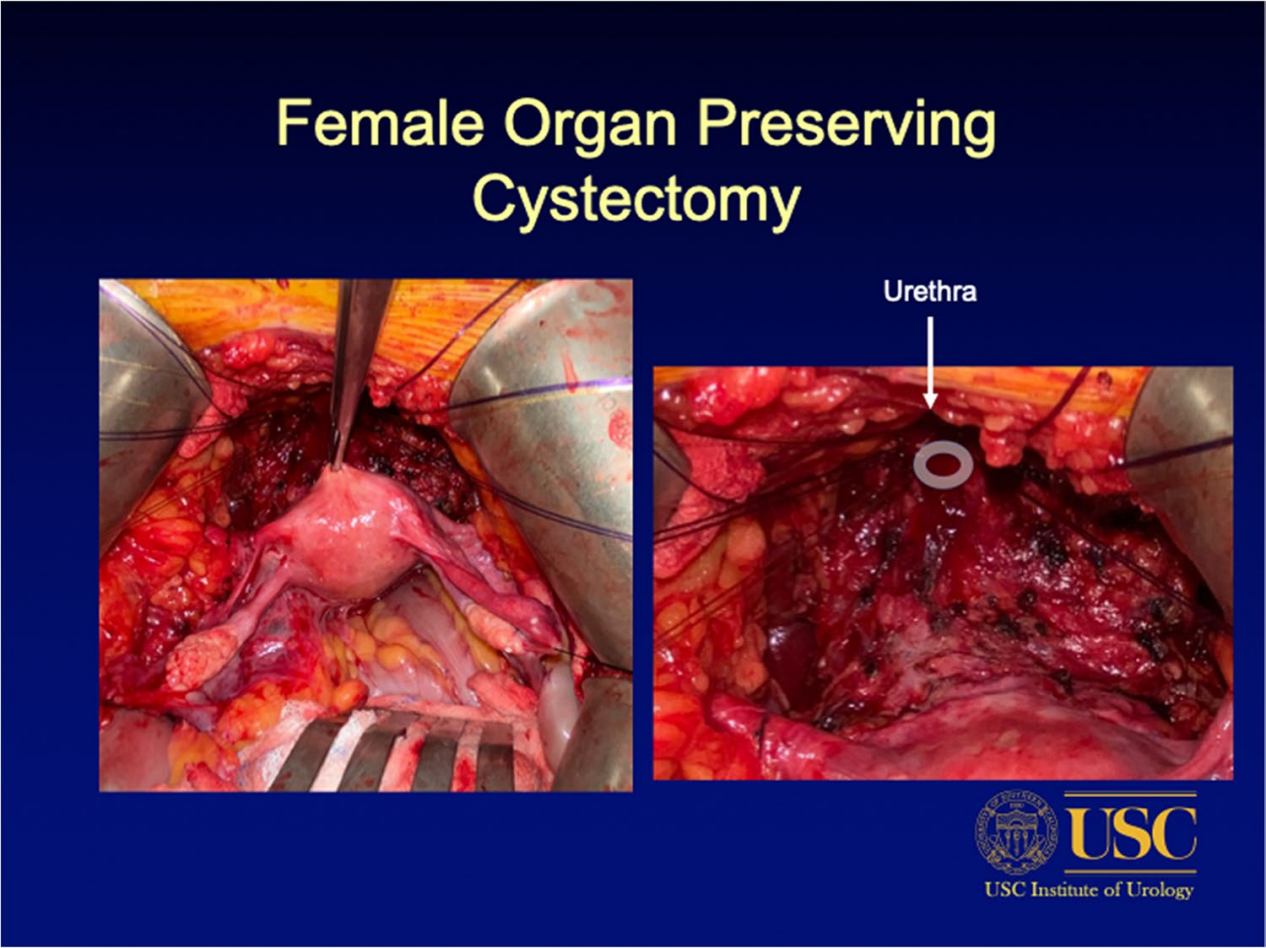
Pelvic organ-sparing radical cystectomy. Courtesy of Sia Daneshmand, MD

Modifications of the classic female radical cystectomy with anterior pelvic exenteration that were intended to improve functional outcomes have been found to have oncologically equivalent outcomes to the maximally extirpative approach in the appropriately selected patient [47, 48]. These modified techniques include urethral sparing, vaginal-sparing cystectomy, gynecologic or female organ-sparing cystectomy, and nerve-sparing [22, 26, 27, 40, 47, 49–52]. Aspects of these modifications can be directly linked to the anatomical consideration that contributes to functional outcomes. For example, Badawy et al.’s modified uterine-sparing technique outlines a careful dissection of the bladder neck and proximal urethra in order to maintain the pudendal nerve plexus, preserve maximal urethral length, and avoid disruption of endopelvic fascia in order to limit injury to the rhabdosphincter and maintain the integrity of the pelvic floor support [53]. A similar approach is used at our own institution, University of Southern California (Fig. 1). Uterine-sparing approaches have highlighted the importance of organ-sparing in functional outcomes. For example, preservation of the anterior vaginal wall maintains the pubocervical fascia and cardinal ligaments which maintains pelvic support and has been linked to lower rates of incontinence [8, 27]. The Mayo group, which performs vaginal-sparing female cystectomy, found a continence rate of 92% [44]. Preservation or restoration of the vesicopubic-urethro-pelvic ligaments has also been advocated as a means of supporting the urethra and distal vaginal wall and thereby improving continence outcomes [54].

Aside from the objective outcomes of improving continence and reducing the likelihood of pelvic organ prolapse, improved functional outcomes have also been linked to quality-of-life outcomes for female cystectomy patients with ONBs. Several studies have highlighted the importance of continence, voiding function and voiding symptoms to overall quality of life among female cystectomy patients [44, 45, 55, 56]. The generalizability of much of the literature on functional outcomes has been limited by the lack of validated questionnaires assessing quality of life and integrating urinary symptoms into quality-of-life assessments for female cystectomy patients. Questionnaires include the Bladder Cancer Index (BCI) from the University of Michigan, CONTILIFE and the Functional Assessment of Cancer Therapy-Bladder Questionnaire [57–59]. Bartsch et al. demonstrated that women with neobladders prioritize dryness over spontaneous voiding as these women were more bothered by nighttime incontinence than they were with having to perform clean intermittent catheterization (CIC). In addition to using the Michigan Bladder Cancer Index, female patients with neobladders were asked whether they would rather perform CIC or leak urine, with 66% of patients with preference for CIC of which 63% of those performing CIC stated they are not bothered by it [58]. The seeming preference for CIC over incontinence points to the nuances of counseling regarding voiding and incontinence after female cystectomy with neobladder.

## Continence and the Female Orthotopic Neobladder

Continence for female patients who have undergone cystectomy with ONB is a dynamic entity that includes assessments of frequency, severity, and temporality of urinary leakage. Long-term analysis of functional outcomes has demonstrated the evolution of continence status over time with some patients gaining continence and others developing the inability to urinate, or hypercontinence, after the immediate post-operative period [44, 58]. Similar to discussions of female urinary incontinence in the benign literature, there is significant variability in the definition of continence among female cystectomy patients with neobladders. Continence can be defined either based on pad use or on assessments of the degree of dryness. Literature regarding the nerve-sparing technique for the female cystectomy, for example, overwhelmingly relies on “zero pad use” as the definition of continence [38, 44–46, 51]. A more subjective definition of continence that has been used is based on “frequent leakage or no urinary control whatsoever” [10, 31]. Intraoperative factors specifically linked to worsened continence outcomes include history of prior concomitant hysterectomy, lack of nerve-sparing technique, shorter functional urethral length and urodynamic demonstration of a lower maximum urethral pressure [17, 55, 60–64].

Questionnaires that have been designed to assess urinary symptoms and health-related quality of life among bladder cancer patients such as University of Michigan’s BCI have only been validated in male patients [65]. Its use in evaluating urinary continence and its contribution to quality of life among female bladder cancer patients has been adapted and neglects to account for differences in the experiences of continence between male and female cystectomy patients [58]. To date, there are no validated questionnaires to assess continence in a female cystectomy patient population with ONBs. As quality of life is more closely linked to continence symptoms, the development of validated instruments for women with ONBs after cystectomy is a necessary next step.

Functional outcomes following cystectomy have centered on determination and evaluation of continence during the day and at night [38, 44, 45, 55, 66]. The characterization of incontinence in bladder cancer patients with ONB is based on three categories that are informed by the differential mechanisms thought to contribute to the type of incontinence/retention that can be seen: daytime incontinence, nighttime incontinence and hypercontinence or urinary retention (Table 1).

**Table 1:** Types and Etiologies of Lower Urinary Tract Symptoms in Female Patients with Orthotopic Neobladder

**Type of Incontinence**	**Proposed mechanisms**	**Risk factors**
Daytime incontinence	Lack of pelvic floor support	Prior SUI, older age, lack of pelvic-organ sparring
Nighttime incontinence	Decreased reservoir capacity	Patient age, medical comorbidities
	Rhabdosphincter relaxation during sleep	
	Nocturnal overdistension of the neobladder with failure of urethral closure	
	Physiologic diuresis	
Hypercontinence	Excess of sympathetic tone preventing appropriate sphincteric relaxation	Non-nerve-sparing approach, pelvic exenteration, post-op chemotherapy, nodal involvement, concomitant hysterectomy
	Folding of atonic proximal urethra	

## Pelvic Floor Support and Post-Cystectomy Incontinence

Lack of pelvic floor support is thought to contribute to daytime incontinence in the post-cystectomy neobladder female patients, occurring via similar mechanisms as those that predispose women to stress urinary incontinence (SUI) in the benign context [55, 61]. The correlation between SUI and daytime incontinence in female patients with neobladders is further evidenced by the similarity in risk factors for daytime incontinence, as history of vaginal delivery is a shared risk factor. Daytime incontinence was also found to be more severe among those with SUI prior to cystectomy, among those with prior hysterectomy, and among older patients [56, 57]. Additionally, when more pelvic support is preserved at the time of cystectomy, daytime incontinence rates improve [17, 26, 27, 67]. Overall daytime continence rates for female bladder cancer patients that have undergone cystectomy with ONB range from 64 to 90% [38, 52]. Higher rates of daytime continence are observed among women undergoing pelvic organ-sparing radical cystectomy, with rates ranging from 80 to 100% [17, 49]. The Mayo group reported a 90% daytime continence rate (defined as completely dry) after a pelvic organ-sparing approach, with two patients achieving daytime continence with urethral bulking agents [44]. Conversely, lower continence rates are reported in women who had undergone anterior pelvic exenteration (65%) and vaginal-sparing surgery (64%), further reinforcing the relationship between surgical approach and preservation of pelvic support and continence outcomes [8, 12, 52, 58, 66].

Heterogeneity exists in the distribution of incontinence type among female cystectomy patients with orthotopic neobladders [56]. Patients with daytime incontinence may not necessarily exhibit nighttime incontinence and vice versa. The lack of a connection between the occurrence of nighttime and daytime incontinence points to a distinct mechanism predisposing female cystectomy neobladder patients to nighttime incontinence. As an isolated phenomenon, nighttime incontinence is more closely related to reservoir capacity. As such, patients with an orthotopic neobladder can note improvement in their nighttime incontinence over time as the functional capacity of the neobladder improves [38, 61]. Other posited mechanisms for nighttime incontinence include rhabdosphincter relaxation during sleep, nocturnal overdistension of the neobladder with failure of urethral closure, and, similar to nocturia, physiologic diuresis [60]. In addition to considering the possible causes of nighttime incontinence that are unique to the neobladder, it remains important to assess for contributing factors such as obstructive sleep apnea or nocturnal polyuria that may have existed previously and have been unmasked by an orthotopic neobladder.

## Mechanisms of Hypercontinence in the Female Orthotopic Neobladder

Several theories exist regarding the etiology of hypercontinence in the female cystectomy patient with orthotopic urinary diversion. The impact of denervation on hypercontinence has been suggested by two distinct mechanisms. One theory is that denervation of parasympathetic fibers in a non-nerve sparing approach leads to an excess of sympathetic tone preventing appropriate sphincteric relaxation [35, 68]. Yet, the lack of responsiveness of female neobladder patients with urinary retention to alpha-blocker therapy suggests that excessive tone is not the only contributor to urinary retention in female neobladder patients. Otherwise, complete denervation that occurs in more extensive resection can result in an atonic proximal urethra that may fold during voiding, resulting in obstruction [60]. While this mechanism has been supported by the radical hysterectomy literature, it has not been demonstrated mechanistically in animal studies [35, 69]. Case series have identified receipt of post-op chemotherapy (HR 4, p = 0.02), nodal involvement (HR = 13, p = 0.02) and pathological stage > T3 (HR 5, p = 0.002) as risk factors for hypercontinence [52]. As all of these factors are associated with more advanced disease, it is certainly possible that intraoperative decisions based on disease status that tend towards more extensive resection (e.g., hysterectomy, lack of nerve-sparing) are driving the relationship between these factors and the risk of hypercontinence. Other possible causes of hypercontinence include sphincter dyssynergia or an overcapacious reservoir preventing adequate emptying [60].

As with all aspects of functional outcomes in female cystectomy patients with orthotopic neobladder, the surgical approach informs the likelihood of developing hypercontinence. At the University of Southern California, they compared the continence outcomes across different surgical approaches [52, 58]. Hypercontinence rates were observed in 33% of all 57 patients and were higher amongst patient undergoing orthotopic neobladder in combination with anterior pelvic exenteration (63%) as compared to vaginal-sparing technique (31.5%) [52, 58]. As observed in other series, hypercontinence developed over time with patients taking anywhere from 7 to 16.7 months to develop urinary retention, with a median time of 12 months. In a cohort of patients who had undergone robotic organ-sparing cystectomy with orthotopic ileal neobladder, 50% demonstrated hypercontinence, although hypercontinence was defined as the need for clean intermittent catheterization (CIC) which has also been recommended for management of nighttime incontinence [38].

Another factor thought to contribute directly to hypercontinence in the female cystectomy neobladder patient is the presence of pelvic organ prolapse (POP). POP in the female cystectomy patient is thought to contribute to hypercontinence by causing direct urethral kinking at the neobladder-urethra anastomosis [70]. Dynamic MRI and videourodynamic studies have provided evidence in support of the relationship between neobladder orientation and voiding dysfunction in the female orthotopic neobladder patient [55, 71, 72]. Women with urinary retention were found to have a pouchocele and the absence of neobladder prolapse was associated with more complete neobladder emptying with voiding [72]. In further support of this theory, analysis of voiding dysfunction after round ligament fixation of the neobladder found that rates of elevated PVR were lower in those with round ligament fixation compared to standard cystectomy with neobladder (23.1% vs 45.9%) and a lower likelihood of needing to perform CIC (10.3% vs 40.5%, HR = 0.22) [73]. POP in the neobladder patient has similarities to its benign corollary with respect to risk factors—lack of pelvic support, history of concomitant or prior hysterectomy—yet differs significantly with respect to the management due to the absence of normal female anatomic structures. The true prevalence of POP amongst women with bladder cancer who have undergone cystectomy with neobladder is unknown due to underreporting. However, most women with POP in the setting of a neobladder have apical prolapse due to the loss of support [53, 70, 74]. A large case series by Badawy et al. reported an incidence of 6% of POP in women with ONB after a follow-up period of 62 months and found the rate of vaginal prolapse after cystectomy to be 6% with ten years of follow-up [53].

Additionally, the relationship between POP and hypercontinence in female neobladder patients is thought to be bi-directional. The presence of POP pre-operatively is an important factor in terms of the development of hypercontinence [75]. Hypercontinence itself is thought to be a risk factor for POP development as frequent Valsalva efforts in a pelvic floor with disrupted support can lead to a neocystocele or enterocele with downward migration of the neobladder-urethra angle [70, 76]. In rare instances, vaginal cuff dehiscence with complete evisceration can occur [77].

## Prophylactic Techniques and Functional Outcomes

While there are no absolute guidelines recommending prophylaxis for POP at the time of cystectomy and orthotopic neobladder, prophylactic procedures have been incorporated into many practices. The focus of many of the prophylactic procedures is to provide or reinforce posterior support to the neobladder due to the theorized relationship between the alignment of the neobladder-urethral angle and the risk of voiding dysfunction in order to decrease the likelihood of aberrant orientation leading to hypercontinence [64]. Posterior support is secured either by suspension of the apex or of the posterior wall specifically including fixation of the omentum anteriorly and laterally to the endopelvic fascia at the time of cystectomy [53], or with a peritoneal bolster sutured to the vaginal stump [78] and the neobladder being affixed to the rectus muscle [17]. The USC group was the first to describe concurrent abdominal sacrocolpopexy at the time of radical cystectomy with orthotopic neobladder with the use of Marlex mesh, with 33% of patient requiring intermittent catheterization, and with other groups following suit [79]. In the series by Stearns et al. at 59.1 months follow-up, there was no reported hypercontinence, mesh erosion or prolapse [54]. Prolapse as the contributor to hypercontinence is further illustrated by the improved decrease in rates of urinary retention in neobladder patients who underwent concomitant sacrocolpopexy at the time of cystectomy [80]. Abdominal sacrospinous ligament fixation has been described with a laparoscopic approach using mesh [81]. More recently, the round ligament has been identified as an anchor point for improving posterior support of the neobladder in which the round ligaments are preserved and anchored laterally to the neobladder in order to provide hammock-like posterior support to prevent downward migration of the neobladder [73].

## Post-Cystectomy Neobladder and Urinary Fistula

Evaluation of urinary incontinence in a female patient following cystectomy with orthotopic neobladder should include consideration for urinary fistula once other possible sources have been excluded. Urinary tract fistulas are often an unrecognized cause of urinary incontinence. In a case series of female orthotopic neobladder patients from a single center, half of the 12 patients were found to have undiscovered urinary tract fistula [82]. Similar to other functional outcomes following radical cystectomy, reduction in the degree of resection improves outcomes with respect to risk of fistula as lower rates of urinary tract fistula were observed when a vaginal-sparing technique was used [8, 17, 83]. Of the patients in the Mayo group, only 5% developed a neobladder-urinary tract fistula with one patient requiring conversion to an ileal conduit due to fistula recurrence [44]. As determined by the USC group, the location of the neobladder-vaginal fistula impacted the likelihood of both successful repair and the development of urinary incontinence following repair as improved outcomes were noted when fistulas were located in the anterior vaginal wall as compared to near the urethra-neobladder anastomosis (84).

## Conclusion

Overall, functional outcomes of women with bladder cancer undergoing racial cystectomy with orthotopic neobladder diversion are understudied. Significant differences in anatomy warrant a distinct evaluation of functional outcomes following radical cystectomy with orthotopic neobladder in female patients to allow us to better understand what outcomes are important and are potential targets for optimizing options. A better appreciation of functional outcomes for the female orthotopic neobladder is also important in guiding decision-making around treatments. However, data in the field is limited by small sample sizes and inconsistencies with respect to definitions of outcomes. To that end, the development of validated questionnaires specifically designed to assess functional outcomes in female bladder cancer patients with orthotopic neobladder will help to resolve inconsistencies in reporting outcomes, allowing for differential tracking based on surgical approach or technique and will permit clear quantification of symptom severity and bother. With a heightened focus on survivorship for cancer patients, furthering our understanding of this understudied patient population will enable us to provide higher quality care, much of it rooted in the tenets of female reconstructive surgical technique.

## Data Availability

No datasets were generated or analysed during the current study.
